# Neuronal delivery of nanoparticles via nerve fibres in the skin

**DOI:** 10.1038/s41598-021-81995-x

**Published:** 2021-01-28

**Authors:** Neeraj Katiyar, Gayathri Raju, Pallavi Madhusudanan, Vignesh Gopalakrishnan-Prema, Sahadev A. Shankarappa

**Affiliations:** Center for Nanosciences and Molecular Medicine, Amrita Institute of Medical Sciences and Research Center, Amrita Vishwa Vidyapeetham University, Kochi, 682041 Kerala India

**Keywords:** Cell delivery, Nanoparticles, Somatic system

## Abstract

Accessing the peripheral nervous system (PNS) by topically applied nanoparticles is a simple and novel approach with clinical applications in several PNS disorders. Skin is richly innervated by long peripheral axons that arise from cell bodies located distally within ganglia. In this study we attempt to target dorsal root ganglia (DRG) neurons, via their axons by topical application of lectin-functionalized gold nanoparticles (IB4-AuNP). In vitro, 140.2 ± 1.9 nm IB4-AuNP were found to bind both axons and cell bodies of DRG neurons, and AuNP applied at the axonal terminals were found to translocate to the cell bodies. Topical application of IB4-AuNP on rat hind-paw resulted in accumulation of three to fourfold higher AuNP in lumbar DRG than in contralateral control DRGs. Results from this study clearly suggest that topically applied nanoparticles with neurotropic targeting ligands can be utilized for delivering nanoparticles to neuronal cell bodies via axonal transport mechanisms.

## Introduction

Neurons of the peripheral nervous system are involved in the processing and propagation of electrical signals to and from the central nervous system. Strategies to deliver therapeutic molecules to the peripheral neurons have the potential to be used in several clinical conditions, such as neuropathic pain^1^, neuropathies^2^, nerve injury^3^, and regeneration^4,5^. The cell bodies of peripheral neurons give out axons that form distinct bundles within nerves, and innervate limbs, organs, and other tissues. Although axons are an important therapeutic target for various nerve disorders, the neuronal cell body itself can also be potentially targeted for alteration of gene and protein expression.

Attempts to deliver drug molecules to the peripheral neurons has been difficult, primarily because of the restrictive neuroanatomical distribution of the peripheral nerves^6^, and also due to the presence of the tight blood-nerve barrier that protects the nerve endoneurial microenvironment. Systemic route of drug delivery is associated with high drug concentration related off-target effects, while local delivery around a nerve, as used in regional and nerve block anaesthesia, are quite limited in their application^7^.

To circumvent many of these limitations related to nerve-drug delivery, nanoparticles have been utilized to specifically target neural tissue^8,9^. Attempts to deliver specialized nanoparticles of various sizes and surface decorations to neural tissue have yielded mixed results^10,11^. Though nanoparticles are small enough to be passively transported through most cell junctions^12^, the blood-nerve barrier still limits nanoparticle entry^13^. Direct administration of nanoparticles locally around a peripheral nerve has been shown to result in partial entry of nanoparticles into the nerve substance^14^, though their distribution specifically within axons and cell body is not known. Additionally, direct application of nanoparticles around the nerve requires expertise in neuroanatomical location of the nerve and the procedure itself can be technically challenging. In the midst of these limitations, a possible alternative strategy to access peripheral axons and consequently the neuronal cell bodies, could be from the skin. The skin is innervated by a vast number of axonal terminals, which are the branched endings of peripheral nerves. Several neurotropic viruses, such as the rabies virus, are known to utilize peripheral axons to gain access into individual neurons and translocate themselves to neuronal cell bodies, and even to the brain^15–18^. In this study, we took motivation from such neurotropic viruses and attempted to deliver gold nanoparticles via skin nerve endings to neuronal cell bodies. We and others have previously reported that topically applied metallic nanoparticles not only penetrate skin layers, but also tend to aggregate in the nerve fibre rich, epidermal-dermal junction^19–21^. Here we demonstrate that the epidermal-dermal fibres of sensory neurons can be used to deliver nanoparticles to their respective cell bodies, which potentially has wide applications in treating conditions such as neuropathic pain and other neuropathies.

## Results

### Surface functionalization and characterization of gold nanoparticles

**Figure 1:**
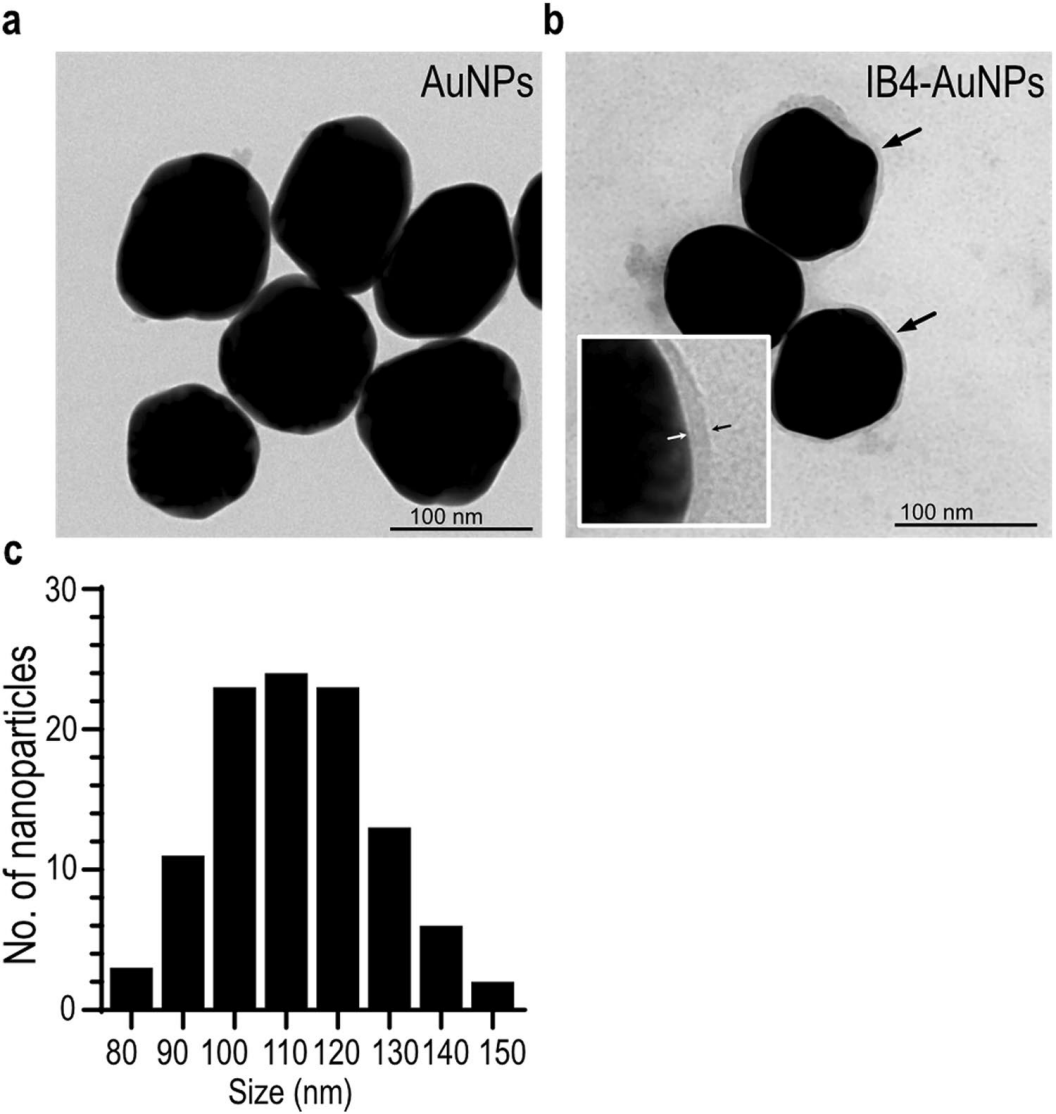
Surface functionalization of AuNP with IB4. Representative transmission electron microscopy (TEM) images of ‘as-synthesized’ citrate capped AuNP **(a)** and IB4-AuNP **(b)**. Arrows indicate a corona on AuNP surface (inset), suggestive of IB4 adsorption. Histogram **(c)** depicting AuNP diameter quantified from 15 non-overlapping TEM micrographs (n = 105 particles).

Gold nanoparticles (AuNP) were synthesized by kinetically controlled step-wise seeded growth method, and surface functionalized with isolectin B4 (IB4) through ionic interaction. High resolution TEM images of AuNP showed spherical shaped particles, with an average size of 111.3 ± 14.9 nm (Fig. 1a,c). IB4 functionalized AuNP showed a distinct peripheral corona, suggestive of IB4 adsorption on the surface (Fig. 1b). Hydrodynamic size of AuNP, as measured using dynamic light scattering was 105.3 ± 1.0 nm, and was consistent with the particle size measurement obtained using TEM. Surface adsorption of IB4 on AuNP resulted in an increase in the hydrodynamic size to 140.2 ± 1.9 nm, and decreased the surface charge of AuNP from − 43.3 ± 0.8 mV, to − 24.9 ± 1.9 mV. In addition, IB4-AuNP demonstrated a small red-shift (~ 4 nm) in the surface plasmon resonance (SPR) absorption peak (Table 1), as compared to the peak obtained from non-functionalized AuNP (Supplementary figure S1), further confirming IB4 surface adsorption. Further, estimation of IB4 content on IB4-AuNPs yielded results that suggested conjugation of 7–9 IB4 molecules per AuNP (Supplementary figure S2).

**Table 1:** Gold nanoparticle characterization.

	AuNP	IB4-AuNP
Hydrodynamic size (nm)	105.3 ± 1	140.2 ± 1.9
Poly dispersity index	0.1	0.2
Zeta potential (− mV)	43.3 ± 0.8	24.5 ± 3.4
SPR (nm)	562.8 ± 0.6	566.2 ± 0.2

Data obtained from dynamic light scattering and spectrophotometric analysis of non-functionalized and IB4 functionalized AuNP. Data shown are mean ± standard deviation (S.D.), from three experimental replicates.

### IB4-AuNP bind to cell bodies of dissociated sensory neurons

**Figure 2:**
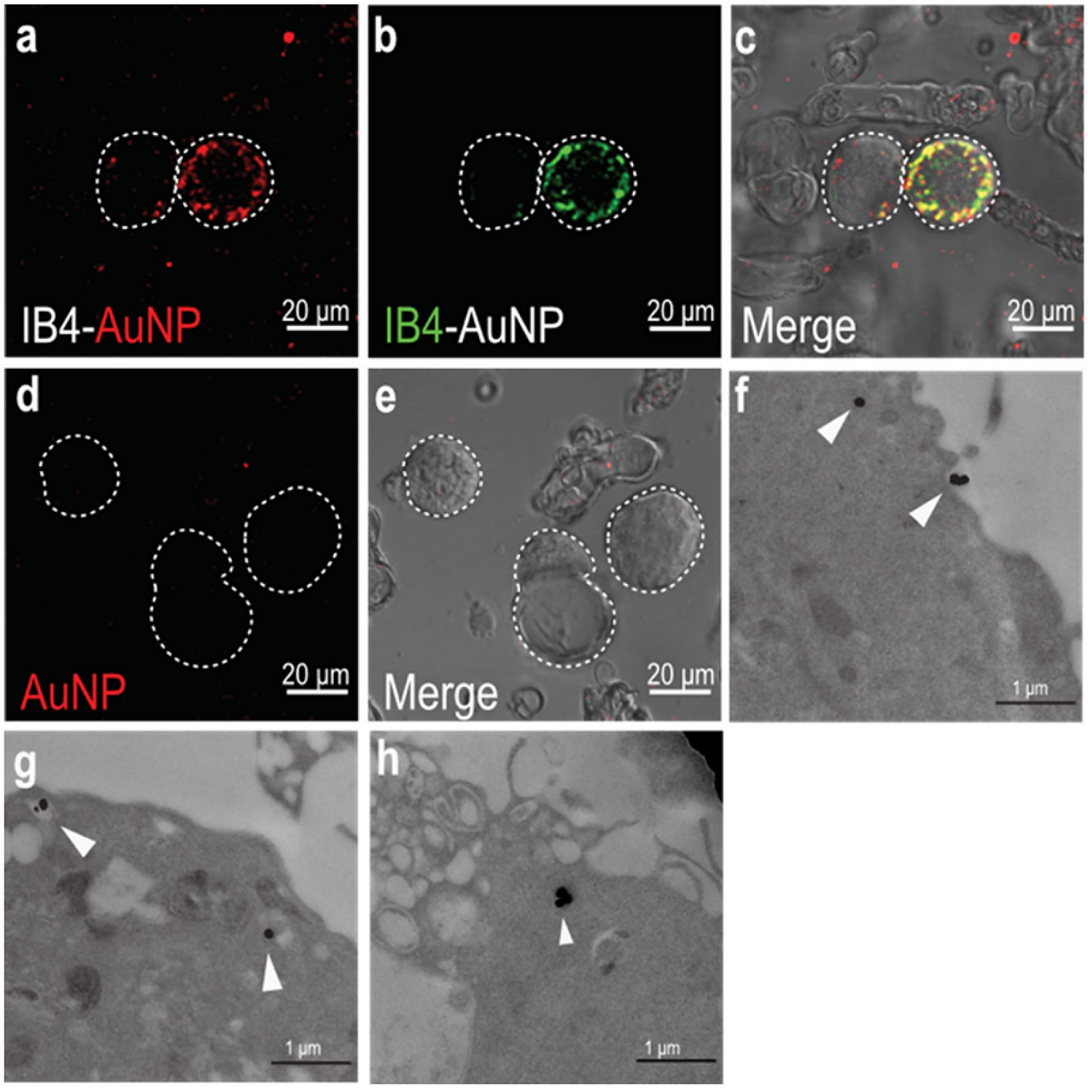
IB4-AuNP bind and enter cell bodies of dissociated sensory neurons. Representative confocal microscopy images of dissociated rat DRG neurons treated with FITC-tagged IB4-AuNPs **(a–c)** and non-functionalized AuNPs **(d,e)**. AuNP associated laser excited light-scattering signals **(a)** and FITC-IB4 associated signals **(b)**, were seen only in specific neurons, while absent in others, indicating IB4 selectivity. Representative TEM images of gold nanoparticles **(f)** binding to DRG cell membrane, and undergoing endocytosis, with nanoparticles localized in well-formed endosome (**g,h** arrows).

To determine neuronal binding and uptake of IB4-AuNP within cell bodies of sensory neurons, dissociated rat dorsal root ganglion (DRG) cell cultures were exposed to varying dilutions of IB4-AuNP. We utilized the SPR-enhanced light scattering property of AuNP to detect optical reflective intensity signals under a laser scanning confocal microscope^22^. Approximately 70–80% of DRG neurons demonstrated strong IB4 binding that was quite distinctly visible on neuronal cell membrane (Supplementary figure S3). In addition, out of the total IB4 positive neurons, 80% were small-diameter neurons, while the remaining were medium and large diameter neurons (Supplementary figure S3). These binding characteristics, to a large extent, determined our choice to use IB4 as the neurotropic ligand. DRG cells exposed to AuNP that were functionalized with FITC-IB4, demonstrated colocalized signals that were clearly observed on the plasma membrane and within the DRG neuronal cell body (Fig. 2a–c), suggestive of binding and uptake of IB4-AuNP. Expectedly, few DRG neurons exhibited no AuNP associated signals (Fig. 2a–c), that was strongly suggestive of IB4-AuNP selectivity. The reflective intensity signals from IB4-AuNP appeared clustered and were not uniformly distributed within the cells. In contrast, cells treated with non-functionalized AuNP (at matched concentration of IB4-AuNP) demonstrated no intracellular AuNP signals (Fig. 2d,e). However, non-functionalized AuNP at higher concentrations show distinct non-specific binding (Supplementary figure S4). To further confirm IB4-AuNP uptake, we obtained TEM images of DRG cells treated with IB4-AuNP. Images obtained after 1 h of IB4-AuNP exposure demonstrated clear surface binding and internalization of the nanoparticles into DRG neurons. Distinct endosomal vacuoles around the internalized nanoparticles were also observed (Fig. 2f–h), suggestive of nanoparticle endocytosis.

### IB4-AuNP bind to spatially separated axons of dissociated sensory neurons

**Figure 3:**
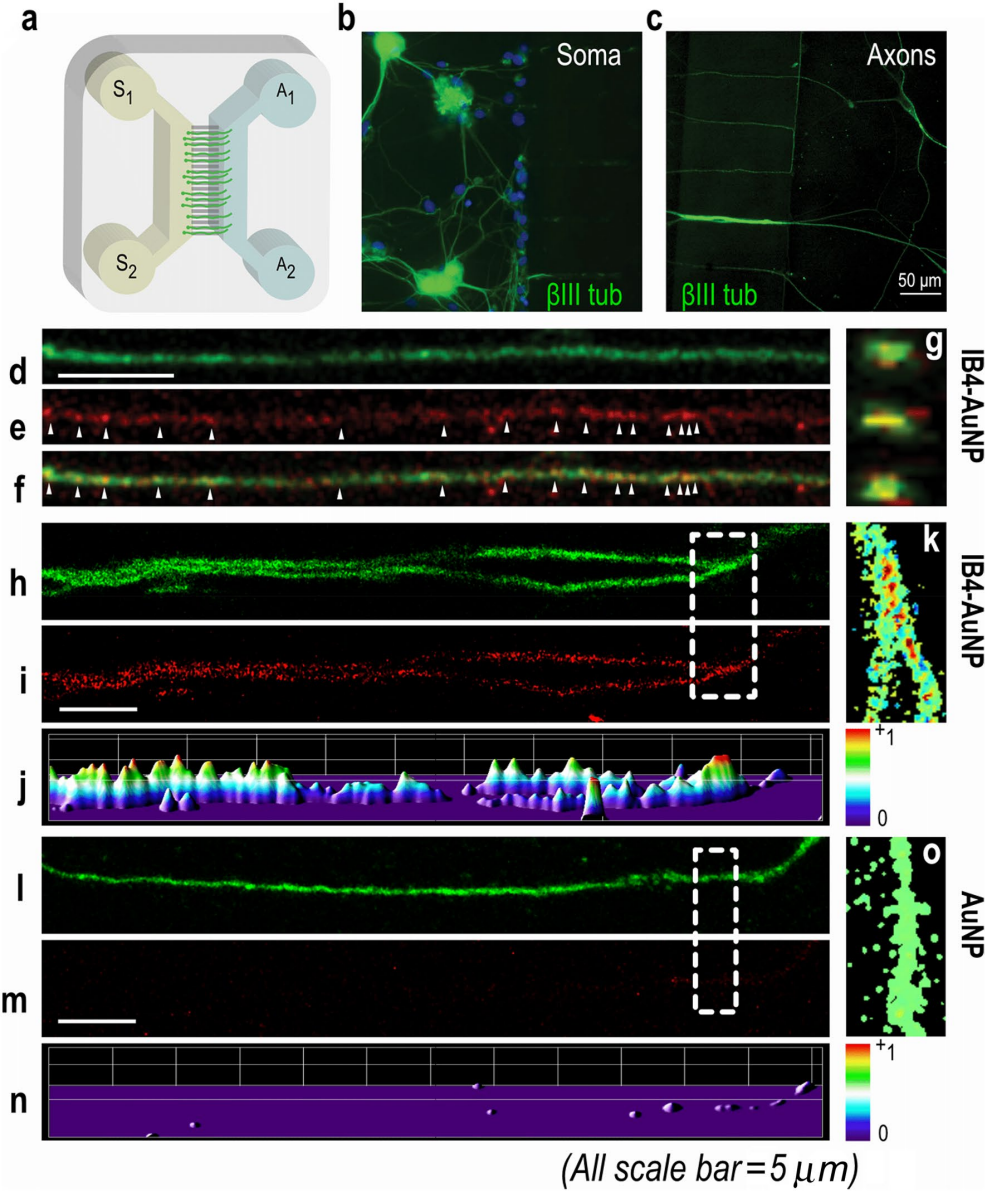
IB4-AuNP binds to axons of dissociated sensory neurons. Schematic representation of a microfluidic device **(a)** showing the soma (s1–2) and axonal (a1–2) compartments, with microgrooves in between. Confocal microscopy images of β-III tubulin stained dissociated rat DRG neurons cultured in the soma compartment **(b)** showing glial cells (blue) and sprouted axons that traversed the microgrooves and were observed in the axonal compartment **(c)**. Representative images of β-III tubulin stained axons **(d)** treated with IB4-AuNP, showing light scattering from AuNP **(e)**. Both signals from **(d)** and **(e)**, co-localized (arrows) at various locations on the axon **(f)**. Optical sections from **(f)**, showing co-localized signals **(g)** from within the axon. Colocalization colour map analysis of randomly selected axonal segments from IB4-AuNP treated devices **(h–k)** showing strong light scatter signals from within axons, while devices with non-functionalized AuNP **(i–o)** showed no light scatter. Experiments were performed in 3–4 devices per group.

To determine axonal binding of IB4-AuNP, neurons cultured in microfluidic system were utilized. Neuronal microfluidic culture system (Fig. 3a) enables the separation of DRG cell soma and axons such that the dissociated DRG cells seeded in the soma compartment extend axons through the microgrooves which emerge in the axonal compartment in 3–4 days. The axonal compartments were then treated with IB4-AuNP in 4-day cultures, and selected axons were imaged using a laser scanning confocal microscope (Fig. 3b,c). All neurons were stained with the neuronal marker, β-III tubulin. Differential fluid levels maintained between the axonal and the soma compartments ensured fluidic isolation within the system and prevented nanoparticle leakage into the microfluidic grooves and the soma compartment (Supplementary figure S5). High resolution images of axons treated with IB4-AuNP demonstrated discontinuous reflective intensity signals along the length of axons, which colocalized well with β-III tubulin signals (Fig. 3d–f). Optical cross-sections of IB4-AuNP treated axons showed strong colocalizing signals in the inner regions of the axons suggesting that the nanoparticles were located within the axons and not just on the surface (Fig. 3g). In addition, the co-localization colour map analysis of specific segments of IB4-AuNP treated axons indicated strong reflective signals from the axons (Fig. 3h–k). In contrast, no signals were detected from axons cultured in microfluidic chambers treated with non-functionalized AuNP (Fig. 3l–o), suggesting that IB4 functionalization is necessary for the uptake of AuNP into sensory axons.

### Axonal transport of IB4-AuNP to the cell body of sensory neurons

**Figure 4:**
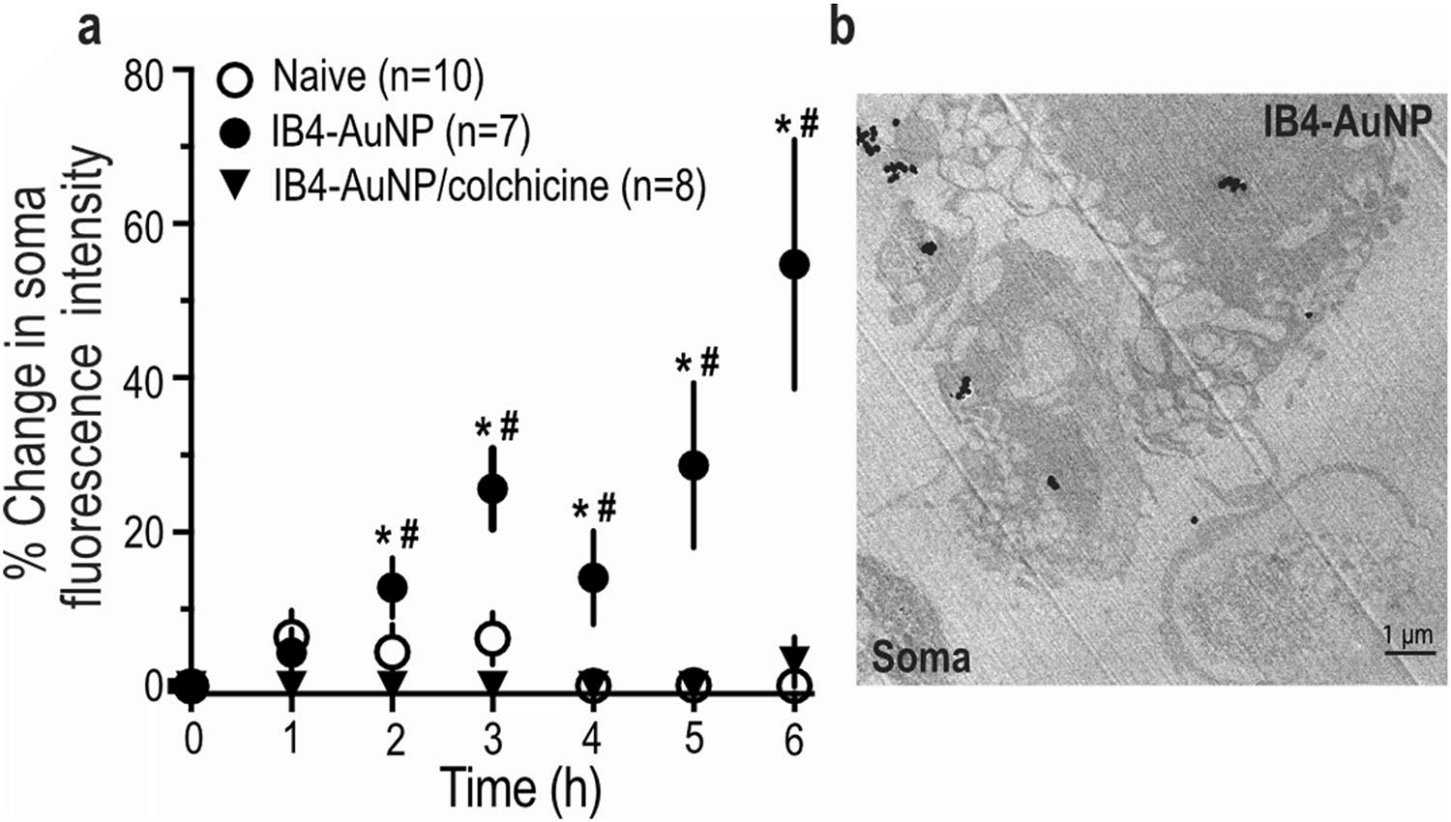
Axonal transport of IB4-AuNP to the cell body of sensory neurons. Graph indicating the time-dependent change in fluorescent intensity from cell bodies of dissociated rat DRG neurons within microfluidic devices, where AuNP conjugated with FITC tagged IB4 were treated in the axonal compartment (a). Data shown are mean ± S.D, * P < 0.05, IB4-AuNP vs Naïve, and # P < 0.05, IB4-AuNP vs IB4-AuNP with colchicine, one-way ANOVA with Sidak’s multiple comparison tests. Representative TEM image of DRG cells **(b)** collected from experiments in **(a)**, after 6 h, showing intracellular localization of gold nanoparticles.

To further determine whether axon bound IB4-AuNP are indeed taken up and transported through axons, we performed time-lapse experiments where change in cell soma fluorescence intensity was measured in the soma compartment after addition of IB4-AuNP in the axonal compartment. Interestingly, we noticed a gradual increase in the DRG soma fluorescence after addition of FITC tagged IB4-AuNP in the axonal compartment (Fig. 4a). The change in fluorescence intensity was less than 20% in the first two hours, but increased drastically after the 3rd hour. Curiously, inhibition of the axonal transport system by colchicine, an inhibitor of microtubule polymerization, prevented the change in soma fluorescence, suggesting that IB4-AuNP translocation to the cell body from the axon, occurs via the axonal retrograde transport system. Naïve neurons showed no changes in cell fluorescence. We further confirmed the translocation of IB4-AuNP by isolating and subjecting the cells from the soma compartment to TEM imaging (Fig. 4b). TEM images of DRG cell bodies showed the presence of IB4-AuNP within the cytoplasm. Taken together, results from these experiments suggest that IB4-AuNP bind to axons of cultured DRG cells and are translocated to the cell body via the microtubule-based retrograde axonal transport system.

### Binding of IB4-AuNP to peripheral nerve fibres in the skin

**Figure 5:**
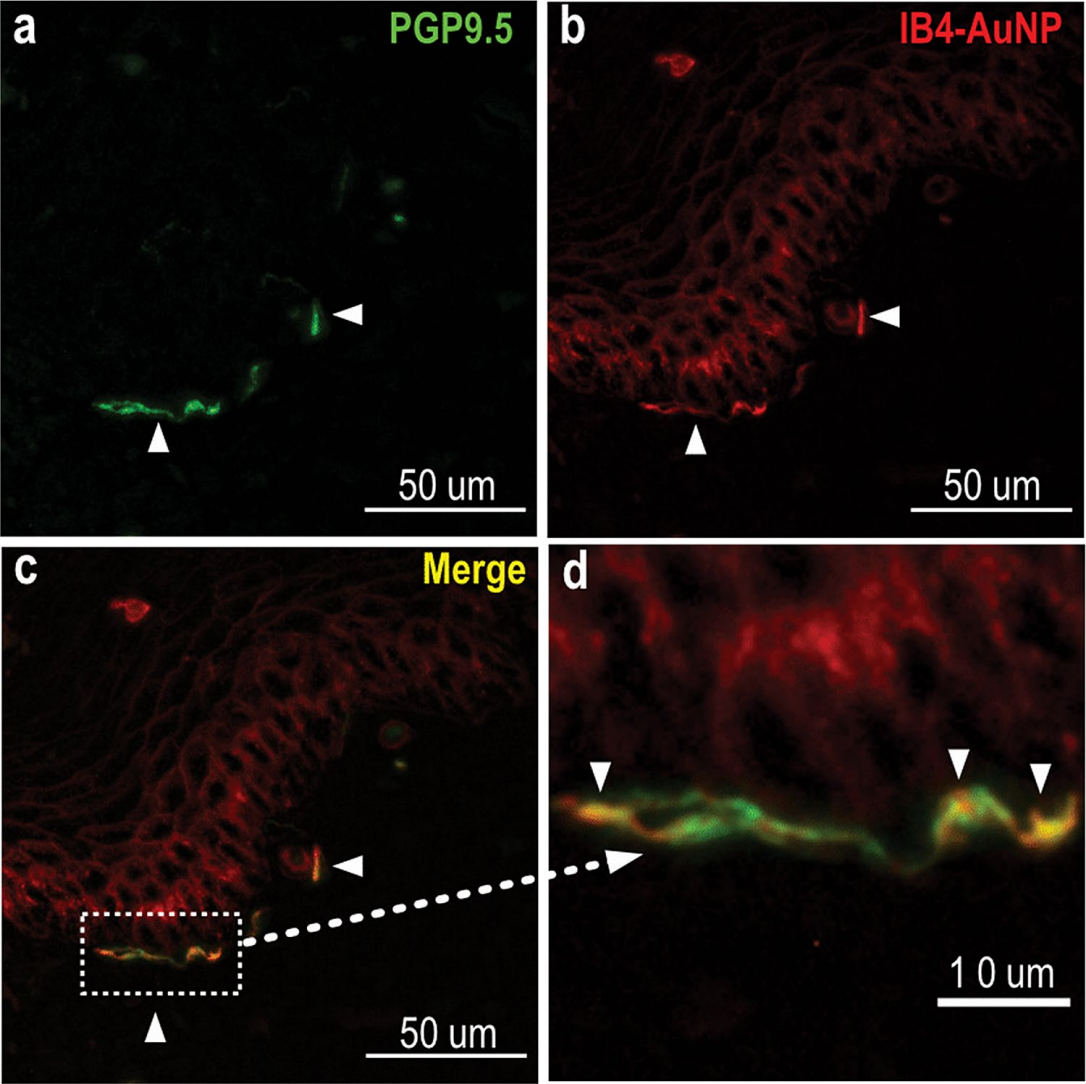
IB4-AuNP binding to peripheral nerve fibres in the skin. Representative fluorescent images of rat hind-paw sections treated with AuNP conjugated with DyLight 594 tagged IB4, and co-stained with anti-PGP 9.5, showing skin nerve fibres **(a)**, and AuNP signals **(b)**, that were found to co-localize **(c,d)**, suggestive of AuNP binding to skin nerve fibres. (n = 3 sections).

To determine binding of IB4-AuNP to terminal axonal endings in skin, we exposed rat hind-paw skin sections to AuNP that were surface functionalized with fluorescently labelled IB4. Skin nerve fibres were identified using the PGP 9.5 protein, which is widely expressed in all nerve tissue. We observed tortuous peripheral nerve fibres, measuring approximately 1–2 µm in diameter, that were distributed within the dermal and epidermal layers of skin (Fig. 5a). Interestingly, fluorescent signals arising from IB4-AuNP colocalized with the dermal and epidermal nerve fibres. Magnified microscopic images distinctly showed that the IB4-AuNP binds to skin nerve fibres in a discontinuous manner, giving an appearance of varicosities along the length of the fibre (Fig. 5a–d).

### Topical application of IB4-AuNP to the rat hind paw

**Figure 6:**
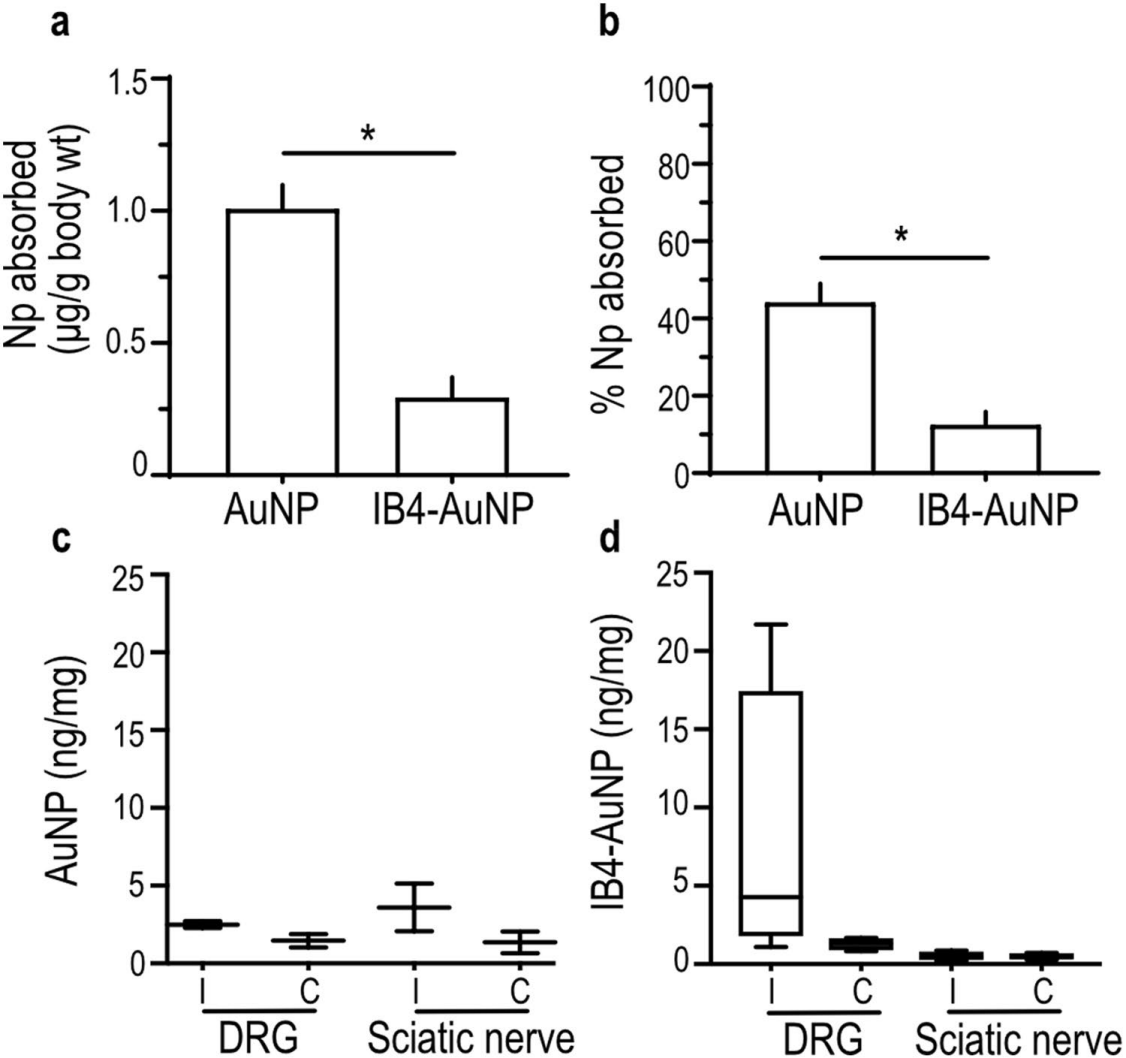
Topical application of IB4-AuNP to the rat hind paw. Bar graphs indicating the amount **(a)** and percent **(b)** of gold nanoparticles that penetrate across the rat hind-paw skin after topical application of non-functionalized AuNP or IB4-AuNP. Data shown are mean ± S.D., with n = 4 rats per group. * P < 0.05, Students t-test. Box-whisker plots indicating the amount of gold nanoparticles detected in DRG and sciatic nerve tissue harvested from rats that were topically treated with non-functionalized AuNP **(c)**, or IB4-AuNP **(d)**. Data shown are measurements obtained from n = 2–4 animal sets per group, with each set comprising of pooled tissue from 4 rats. (i = ipsilateral; c = contralateral).

Binding, uptake, and translocation of topically applied IB4-AuNP in vivo was determined by analysing tissue gold content using inductively coupled plasma mass spectrometer (ICP-MS). The plantar surface of rat hind paw was immersed in solutions containing either IB4 functionalized, or non-functionalized AuNP, at concentrations measuring 187.97 µg/mL and 195.84 µg/mL respectively. The total amount of AuNP that penetrated across skin after 3 h of topical exposure was significantly larger in rats exposed to non-functionalized AuNP, as compared to those exposed to IB4-AuNP (Fig. 6a). Overall, 44.1 ± 4.9% of AuNP were able to penetrate the skin of rats exposed to non-functionalized AuNP in comparison to 12.4 ± 3.4% in rats exposed to IB4-AuNP. Surprisingly, even though the total absorption of nanoparticles was lower in IB4-AuNP treated rats (Fig. 6b), the number of nanoparticles that accumulated in the ipsilateral DRGs of IB4-AuNP group, was much higher compared to the non-functionalized AuNP group (Fig. 6c,d). The contralateral DRGs harvested from IB4-AuNP exposed rats, exhibited nanoparticle numbers that were similar to those harvested from control rats, strongly supporting the possibility that the uptake of nanoparticles observed in the ipsilateral DRG harvested from IB4-AuNP group most likely occurred due to axonal transport from the skin. There was no difference in the gold content measured in sciatic nerve segments harvested from any of the groups.

### Peripheral nerve function of rats exposed to topical AuNP

**Figure 7:**
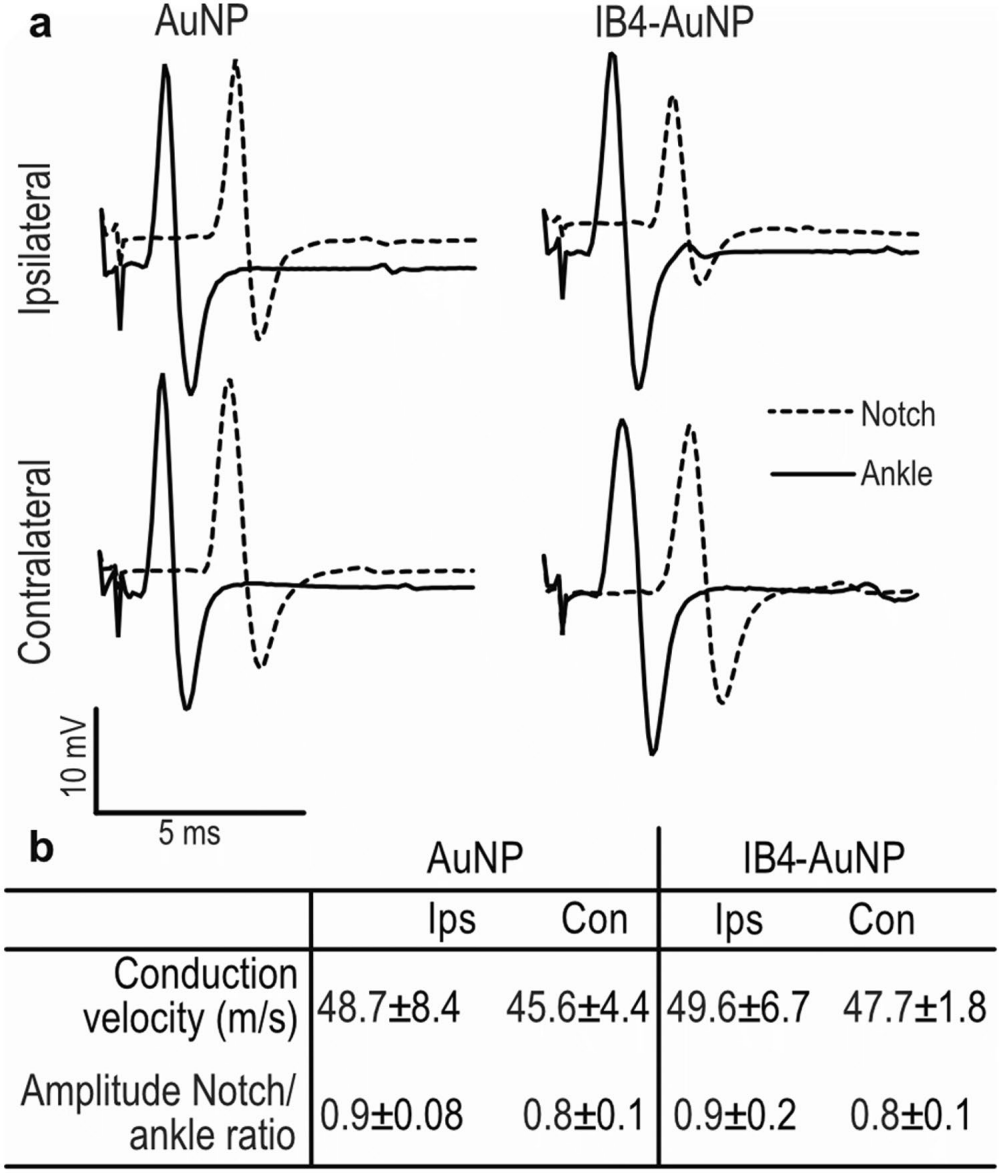
Peripheral sciatic nerve function of rats exposed to topical AuNP. Representative electrophysiological tracings obtained from the plantaris muscle by stimulating the sciatic nerve at the ankle and sciatic notch from AuNP **(a)** and IB4-AuNP treated rats. Conduction velocity and signal amplitudes are shown in **(b)**. Data shown are mean ± S.D., n = 4. (ips = ipsilateral; con = contralateral).

Considering the observation that IB4-AuNP were found to undergo axonal transport, nerve electrophysiological studies were performed to assess nerve function and viability. The conduction velocities recorded from sciatic nerves of rats treated with non-functionalized, or IB4-AuNP were comparable (Fig. 7a,b). Similarly, the obtained peak compound muscle action potential ratio recorded from two different anatomical landmarks in the vicinity of the sciatic nerve did not exhibit any difference between the non-functionalized and IB4-AuNP groups. This suggests that the topically applied IB4-AuNP in the concentration tested did not interfere with peripheral nerve function.

## Discussion

Peripheral nerve fibres innervating the skin are arguably the least explored targets for delivering therapeutic molecules and their carriers. Barring topical anaesthetics that are used for blocking nociceptive receptor activity on peripheral axons^23,24^, clinically there are almost no other drug molecules or delivery systems that target peripheral nerve fibres. In this study, 105.3 ± 1.0 nm sized AuNP were functionalized with IB4 to target axonal terminals and promote binding and uptake. Surface functionalization of AuNP with IB4 resulted in the reduction of total surface charge by almost − 20 mV, and an SPR peak shift that was most likely due to the positively charged lysine and tryptophan rich sites on IB4^25^.

IB4-AuNP were found to bind sensory neurons dissociated from rat DRGs. It is worth noting that IB4 was selected from a list of known neurotropic ligands, including rabies virus glycoprotein (RVG)^26^, tetanus toxin C^27^, and wheat germ agglutinin^28,29^. Though RVG29 has been used to target Neuro 2A cells and mouse brain cells in culture^30^, we were unable to detect RVG29 binding and uptake in DRG derived sensory neurons (data not shown). IB4 was selected based on its selectivity in binding sensory neurons^31^, and for possible future application in pain mitigation. In addition, IB4 has been reported to bind almost 70% of small diameter sensory neurons^32^, which are known to predominately carry nociceptive signals and express pain associated proteins such as Nav1.8, Nav1.9, and TRPV family of receptors^33^. In our study, we observed IB4-AuNP bound to cell membrane of sensory neurons, and additionally found AuNP excitation scatter within neurons, suggestive of uptake. Here, it is important to note that we have utilized the intrinsic property of AuNP to scatter light, for direct visualization under a confocal microscope. It is well-known that at specific wavelengths of incident light, the surface plasmon resonance phenomena of 100 nm sized AuNP causes light to be reflected in the 650–700 nm range^22,34^.

IB4-AuNP were found bound to axonal fibres in the dermal-epidermal regions, and also to a few intra-epidermal nerve fibres in thin skin sections. Individual nerve fibres in the skin measure about 1–4 microns in diameter^35,36^, which allows for the possibility of nanoparticles to bind and enter terminal axons. Previously, we have showed that gold nanoparticles in the size ranges of 20 nm to 180 nm, easily penetrate all layers of thick skin, and appear in the blood stream within 4 days^19^. In addition, topically applied gold nanoparticles accumulate in the nerve fibre rich, epidermal-dermal regions (Supplementary figure S6), thereby significantly increasing the chances of nanoparticle-nerve fibre contact. Our in vitro studies very clearly showed that IB4-AuNP bind axonal terminals and undergo retrograde axonal transport to the cell bodies. Previous in vitro studies show that quantum dots and iron oxide nanoparticles undergo endosome associated transport within axons via the dynein-microtubule system^37,38^. Our results agree quite well with this observation since disruption of microtubule polymerization by colchicine, prevented the axonal transport of AuNP. Interestingly our in vitro data also shows a slight decrease in fluorescence intensity at the 4th hour time-point, and an increase in data variability at subsequent time points. This is curious, because it has been well reported that metallic nanoparticles undergo cellular exocytosis from various cell-lines^39–41^, though such phenomenon has not been reported in primary sensory neurons. It is plausible that the reduction in fluorescence intensity at the soma could be due to differences in the rate of nanoparticle transport and nanoparticle exocytosis. More focused studies are warranted to better explore this phenomenon in differentiated neurons.

Further, our in vivo experiments clearly showed a larger amount of topically applied IB4-AuNP accumulating in the ipsilateral lumbar DRG as compared to the contralateral control. This observation was quite similar to what was observed in cultured axons, where IB4-AuNP accumulated in the cell soma after application at the axonal end. Interestingly, we also observed nanoparticles in sciatic and DRG tissue harvested from rats that were topically exposed to AuNP, albeit in very low amount. Taken together, this data strongly supports the hypothesis that topically applied IB4-AuNP accumulate in the DRG, in large part, by utilizing the axonal innervation from the topical site. Since DRG neurons synapse with second order neurons in the dorsal horn of the spinal cord, it is plausible that topically applied AuNP may translocate to the spinal cord, but this was not checked in this study. Considering our observation that AuNP were detected in contralateral DRG and sciatic nerve, it is possible that part of the total nanoparticles detected in the ipsilateral DRG of rats exposed to IB4-AuNP could also be due to implantation from the systemic circulation. It has been shown that nanoparticles as large as 180 nm can penetrate across intact rat hind-paw skin and appear in the blood stream 4 days later^19^.

Another alternative possibility for IB4-AuNP accumulating in the ipsilateral DRG, could be due to the localized distribution of nanoparticles in the ipsilateral limb, resulting in axonal entry and nanoparticle translocation via nerve fibres innervating the muscle^42^. However, the predominant nerve fibres innervating the muscle tend to be motor with nerve terminals ending in the spinal cord, thus making this route of AuNP delivery less probable.

It is also interesting to note that even though rats were exposed to an almost similar amount of topical AuNP in the IB4-functionalized and non-IB4 functionalized groups, the total amount of IB4-AuNP that were absorbed through the skin was much lower compared to non-functionalized AuNP. This is of much relevance because IB4 functionalization seemed to reduce the number of particles actually crossing the skin, even though the total amount of AuNP accumulating in the ipsilateral DRG was much higher. One probable explanation for reduced permeability of IB4-AuNP compared to citrate capped AuNP could be the uneven surface distribution of lectin on the IB4-AuNP surface. Simulation studies suggest that nanoparticles with homogeneously distributed binding moieties on their surface, similar to our citrate-capped AuNP, dramatically enhance permeability, compared to nanoparticles with random or heterogeneously distributed ligands^43^.

Furthermore, the presence of AuNP within the sciatic nerve in amounts as seen in this study, did not affect nerve function, suggesting the benign nature of this approach. We also did not find any aberrant behavioural alterations that could indicate any sensory dysfunction. However, further safety studies need to be performed to determine functional and cellular effects of nanoparticles within axons.

In conclusion, we have demonstrated that metallic nanoparticles can be specifically translocated to neuronal cell bodies using axonal terminals located in the skin via simple topical application.

## Methods

### Reagents and antibodies

Materials were acquired from the following sources: Gold chloride, poly (ethylene glycol) (PEG), poly-D-lysine (PDL), bovine serum albumin (BSA), colchicine and paraformaldehyde (PFA): Sigma-Aldrich, USA; Trisodium citrate dihydrate, papain, nitric acid, hydrogen peroxide, hydrochloric acid, TRIS-buffer, triton X-100, di-sodium hydrogen phosphate and potassium dihydrogen phosphate: Merck Chemicals, USA; Collagenase-II, dispase-II, fetal bovine serum (FBS), penicillin–streptomycin mix and trypsin–EDTA: Gibco, USA; Hank's buffered saline solution (HBSS), Ham’s F-12 medium and Dulbecco’s modified Eagle’s medium (DMEM): Lonza Chemicals, USA; Isolectin B4, DyLight 594-IB4, FITC-IB4, DyLight 488 horse anti-mouse IgG antibody and Vectashield hardset mounting medium: Vector Laboratories, USA; Glutaraldehyde (EM grade), osmium tetroxide (OsO_4_), sodium cacodylate trihydrate and EMBed-812 embedding kit: Electron Microscopy Sciences, USA; Hoechst 33342: Santa Cruz Biotechnology, Inc. USA; Anti β-III tubulin monoclonal antibody: Cell Signaling Technology, Inc USA; Anti PGP9.5 antibody: Abcam Plc UK; Microfluidic devices (SND 150): Xona Microfluidics, LLC, USA.

### Gold nanoparticle synthesis

Citrate capped AuNP were synthesized using the kinetically controlled seeded growth method as reported earlier^44^. Briefly, 150 ml of trisodium citrate (2.2 mM) was heated to 100 ∘C followed by the addition of 1 mL of gold chloride (25 mM). The solution was maintained at 100 ∘C for 10 min, and then stepped down to 90 ∘C, followed by the addition of 1 mL of 25 mM gold chloride. Additional 1 mL gold chloride (25 mM) was added to the reaction mixture after 30 min. This step was repeated 15–17 times where 55 mL of the reaction mixture was replaced by fresh 1 mL gold chloride (25 mM) and 55 mL of trisodium citrate (2.18 mM) each time, until desired particle size was attained.

### Gold nanoparticle characterization

The hydrodynamic size, dispersity index, and surface charge of synthesized AuNP were determined using Zetasizer Nano ZS (Malvern, UK). UV–vis absorption spectra were measured using Synergy H1 multi-mode plate-reader (Biotek, USA). High-resolution transmission electron microscopy (TEM) images were obtained using the Tecnai G2 F20 TEM (FEI, USA).

### Surface functionalization of gold nanoparticles

IB4 was surface adsorbed on gold nanoparticles as reported earlier^25^. Briefly, 15 µL of IB4 or fluorophore (FITC or DyLight 594) tagged IB4 (1 mg/mL) was suspended in 1 mL of 10 mM phosphate buffer (pH 6.5) containing AuNP, under stirring condition at 4 ∘C. 1% PEG (w/v) was added to the solution, followed by high-speed centrifugation at 400*g* for 30 min and washed thrice. Pelleted AuNP were resuspended in 10 mM phosphate buffer saline (PBS) (pH 7.4) containing 1% PEG (w/v). Surface functionalization was confirmed using absorption peak shift, dynamic light scattering and TEM. It was also observed that non-functionalized AuNPs could be stored stably at 4 °C for up to 6 months, while IB4-AuNPs aggregate within 5–7 days. Hence, only freshly prepared IB4-AuNPs were used in this study.

### Animal care

Adult, female Sprague Dawley (SD) rats weighing 200–250 g were housed in pairs, allowed standard rat diet and water ad libitum, and maintained on 10 h/14 h light/dark cycle. This study was conducted under protocols approved by the Institutional Animal Ethical Committee (IAEC), Amrita Institute of Medical Sciences, Kochi, India (Approval number IAEC/2013/3/5) in accordance with guidelines set forth by the Committee for Control and Supervision of Experiments on Animals (CPCSEA), Government of India.

### DRG neuron isolation and culture

Rats were euthanized by CO_2_ asphyxiation, laminectomy performed and dorsal root ganglia extracted in ice-cold HBSS. DRG neurons were isolated and cultured as reported earlier^45^. Briefly, harvested DRG’s were incubated in papain (> 16 units/mg) for 30 min at 37 ∘C. DRG’s were then transferred to a cocktail of collagenase (4 mg/mL), and dispase (4.66 mg/mL), for 30 min at 37 ∘C, followed by mechanical trituration and suspension in DMEM media containing Ham’s F-12 medium (10% v/v), FBS (10% v/v), and penicillin–streptomycin (1% v/v). For studies involving nanoparticle binding and uptake in cell bodies, freshly isolated DRG cells were exposed to media containing AuNP (10% or 100% v/v) for 20 min, followed by wash, prior to imaging.

### DRG cultures in microfluidic devices

The microfluidic devices were primed as per manufacturer’s instructions. DRG neurons were extracted and dissociated as described above. Approximately 1.5 × 10^6^ cells were seeded in the soma compartment of the microfluidic device. Fluidic isolation was achieved by maintaining a higher volume of media in the soma compartment, as compared to the axonal compartment. Sprouting axons from the soma compartment were observed in the axonal compartment on the 5th day. Nanoparticles were added into the axonal compartment (10 µL, 10% v/v, at 37 ∘C), and washed twice after 20 min before subjecting the devices to imaging studies. In experiments to block retrograde axonal transport, colchicine (10 µM) was added in the axonal compartment for 1 h, prior to nanoparticle treatment.

### Immunostaining studies

Cells in microfluidic devices were washed with PBS (100 mM, pH 7.4) and fixed in PFA (4% w/v) for 25 min, washed and permeabilized with triton X-100 (0.2% v/v in PBS) for 30 min with gentle shaking. Cells were then treated with a blocking solution (BSA, 3% w/v in PBS) for 30 min and incubated overnight at 4 ∘C with mouse anti-β-III Tubulin (1:200) antibody. DyLight 488 tagged horse anti-mouse IgG antibodies were used to detect primary antibody binding, followed by Hoechst nuclear counterstain, and visualized under an inverted confocal laser scanning microscope (DMI6000 CS, Leica Microsystems GmbH, Germany). Non-specific IgG primary antibody was used as controls. Further, immunohistochemical studies were performed on 4% PFA fixed skin sections to determine skin nerve fibre binding of IB4-AuNP. Briefly, 5 µm thin skin sections were permeabilized using Tris-buffer (pH 7.5), and blocked with BSA (1% w/v), for 2 h at RT. Sections were then co-treated with mouse anti-PGP9.5 antibody (1:500), and DyLight 594 tagged IB4-AuNP, overnight at 4 ∘C. Sections were then counterstained with DyLight 488 horse anti-mouse secondary antibody, mounted and visualized under an inverted fluorescence microscope (DMI3000 B, Leica Microsystems, Germany).

### Image acquisition and analysis

For visualizing axonal binding of AuNP, a laser wavelength of 514 nm (argon, 30%) was used to excite the SPR of AuNP under confocal microscopy^22^. All acquisition parameters were maintained at the same settings for all images, and data collected sequentially as multiple image stack files. Light scatter signals from AuNP, along with fluorescent signals from fluorophore tagged antibodies were plotted using Fiji plugin Interactive 3D surface plot v2.4.1. and co-localized as a color map. A randomly selected axonal segment was used to demonstrate level of colocalization, where hot colors represent colocalization of AuNP within axons. For time-lapse imaging experiments, DRG cells in the soma compartment of microfluidic devices were visualized within a live-cell platform. Intensity data log from soma regions were acquired every hour and analysed by Metamorph microscopy automation software (Molecular Devices, USA). Background noise level threshold correction was applied from intensity signals obtained from images acquired at t = 0.

### High-resolution transmission electron microscopy

DRG cells treated with nanoparticles were trypsinized and centrifuged to obtain a pellet, which was fixed with pre-warmed (37 °C) glutaraldehyde (2.5% v/v) in 0.05 M cacodylate buffer (pH 7.4), and post-fixed with OsO_4_ (2% v/v)^46^. Cells in the microfluidic devices were gently scrapped off from the soma compartment and collected by centrifugation. After dehydration, epoxy resin embedded ultra-thin Sections (110–120 nm) were obtained using ultramicrotome (PowerTome PC, RMC Boeckeler Instruments, Inc. USA) and imaged under HR-TEM.

### Topical application of AuNP and distribution

Female SD rats were anesthetized by intraperitoneal administration of ketamine (100 mg/kg) and xylazine (5 mg/kg). The left hind-paw was placed in a modified tube containing nanoparticle solution, such that the plantar area was maximally in contact with the nanoparticle solution. After 3 h, the hind-paw was washed with Milli-Q water (Direct-Q 5 UV, Merck-Millipore, Germany) to remove surface adherent nanoparticles. Care was taken to collect all wash fluid for calculating nanoparticle penetration across skin. Further, we utilized the following equation to determine the amount of nanoparticles (Np) that penetrated across hind-paw skin upon topical application (A_T_).

A_T_ = Total Np amount in solution before topical application − (Amount of Np remaining after topical application + Amount of Np in wash fluid).

Animals were euthanized by CO_2_ inhalation on day 3, and sciatic nerve and lumbar dorsal root ganglion from both sides harvested, and processed for ICP-MS. Tissue samples from 2–3 pooled animal sets per experimental group (each pool containing 4 animals each) were used in the study. Harvested tissue were cut into small pieces and suspended in nitric acid at 80 ∘C for 3 h, and then transferred to a 1:1 mixture of hydrogen peroxide and hydrochloric acid for 1 h. The samples were then diluted with Milli-Q water and analysed using ICP-MS (Thermo Fisher Scientific, Germany).

### Electrophysiological studies

Electrophysiological studies were performed on isoflurane anesthetized rats, 3 days after topical application of IB4-AuNP. Amplitude and latencies obtained from the direct motor response (M wave) and the mono-synaptically evoked H reflex (H wave) were recorded by stimulating the sciatic nerve at the sciatic notch (hip) and the tibial nerve at the ankle. Stimulations were performed via electrodes using a square pulse (5 V, 0.5 ms, 1 Hz) (MP36, BIOPAC Systems, Inc, USA). The recording electrodes were placed in the plantaris muscle of the feet and a reference electrode was placed in the middle digit, and the ground electrode was placed in the inner thigh. Evoked responses were recorded and nerve conduction velocity was calculated by dividing the distance from ankle to notch with the difference in notch—ankle latency values.

### Statistics

All data are shown as mean ± standard deviation (S.D). Difference in values of mean among various experimental groups were statistically tested using appropriate tests as stated in relevant figure legends. Statistical analyses were performed using GraphPad Prism version 7.0.2 for Mac (GraphPad Software Inc, USA).

## Supplementary Information

Supplementary Figures.

## Data Availability

The datasets generated during and/or analysed during the current study are available in the (Mendeley Data) repository (http://dx.doi.org/10.17632/6cj84pw4hn.1).
