# Supplementary material for: Neuronal delivery of nanoparticles via nerve fibres in the skin
Source: Sci Rep. 2021 Jan 28;11:2566. doi: 10.1038/s41598-021-81995-x (PMC7844288; doi:10.1038/s41598-021-81995-x)
Supplement: Supplementary file 1 — Supplementary Figures. [file 41598_2021_81995_MOESM1_ESM.docx]

**Neuronal Delivery of Nanoparticles via Nerve Fibres in the Skin**

Neeraj Katiyar, Gayathri Raju, Pallavi Madhusudanan, Vignesh Gopalakrishnan-Prema, Sahadev A Shankarappa

**Supplementary Information**

**
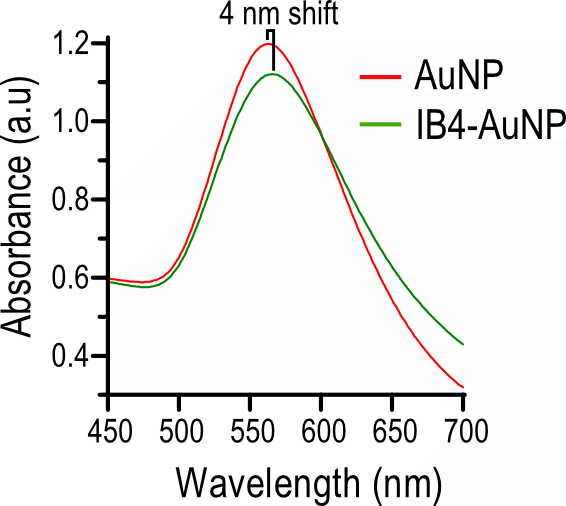
**

**Figure S1:** Representative UV-VIS spectrum obtained from solutions containing non-functionalized and IB4 functionalized AuNP. We observe a 4 nm shift in the IB4-AuNP spectra compared to AuNP spectra, suggestive of IB4 surface adsorption.


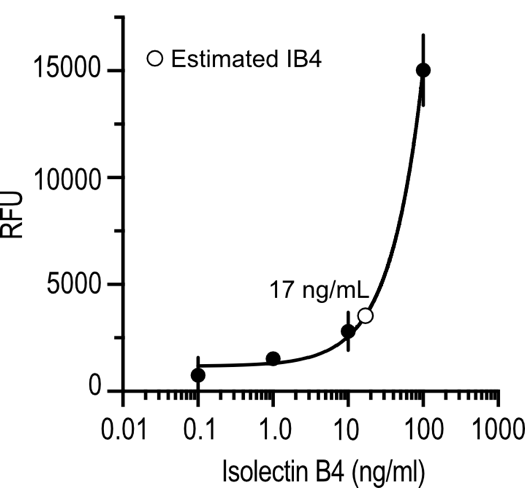


**Figure S2:** Estimation of IB4 content in IB4-AuNP solution. Fluorescence measurements from AuNPs conjugated with FITC-tagged IB4 were compared with a standard curve obtained from free FITC-IB4. Molecular weight of each IB4 molecule (114kDa), was used to estimate the number of IB4 molecules in a solution containing known number of AuNPs (1.2 ± 0.3 x 10^10^ per mL). Based on these results, we estimate that there are 7-9 IB4 molecules per AuNP.


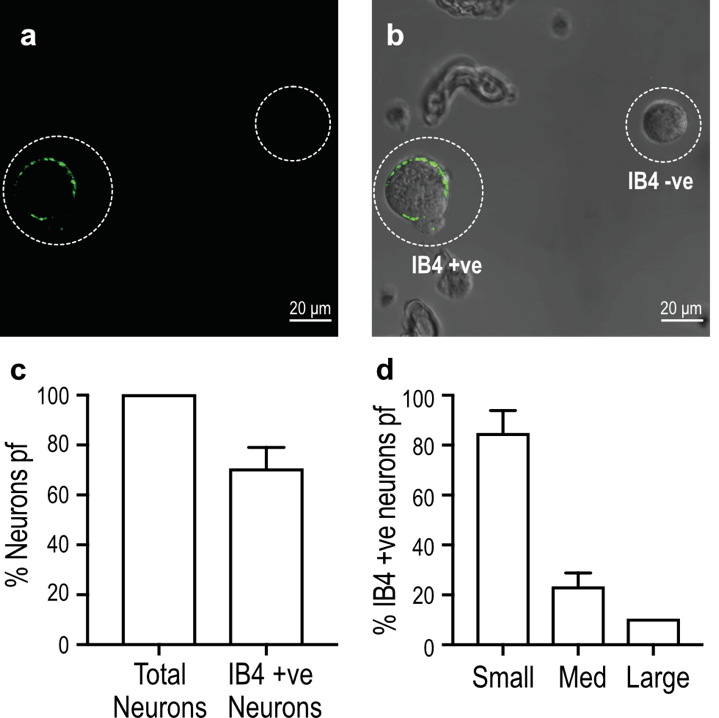


**Figure S3:** IB4-binding in dissociated DRG neurons. Fluorescent images of IB4 positive and negative DRG neurons in (a), and merged bright field image in (b). Quantification of IB4 positive neurons is displayed as bar graphs showing total (c) and cell-size based (d) binding of free-IB4 in DRG neurons per field (pf). Neurons < 25 μm, 25-40 μm, and > 40 μm were categorized as small, medium and large diameter neurons respectively. Data shown are means ± SD from 5-6 images per well (n=5).


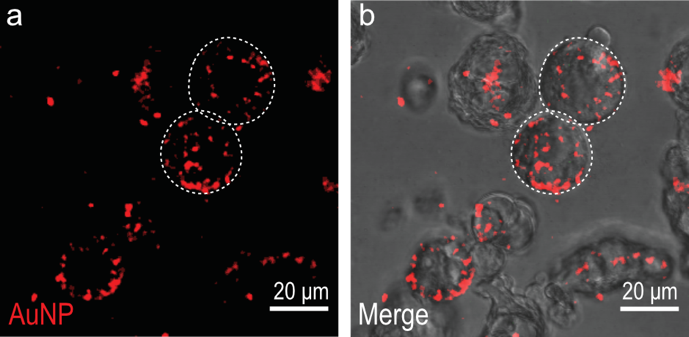


**Figure S4:** Representative confocal images of DRG neurons exposed to high concentration (100% v/v, as-synthesized) of bare-AuNPs. Fluorescent image showing light-scattering from AuNPs in (a), and merged bright field image in (b). Dotted lines indicate DRG neurons showing non-specific binding of AuNPs to small-diameter DRG neurons.

­

**
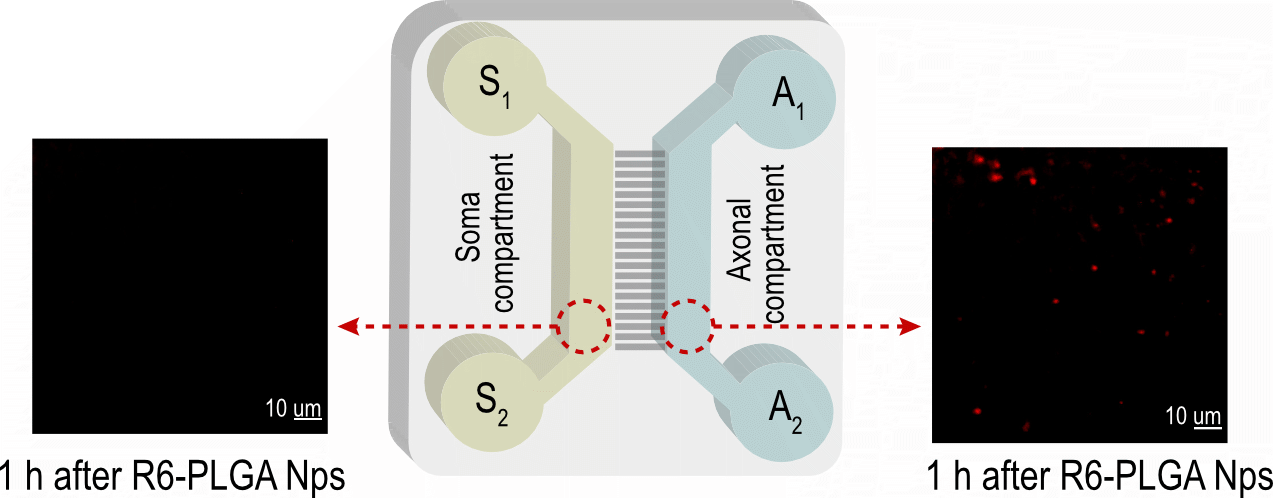
**

**Figure S5:** Representative fluorescence images taken 1 h after addition of rhodamine 6G loaded PLGA nanoparticles (~250 nm) in the axonal compartment. No fluorescence was observed in the soma compartment, confirming the maintenance of fluidic integrity in respective compartments.

**
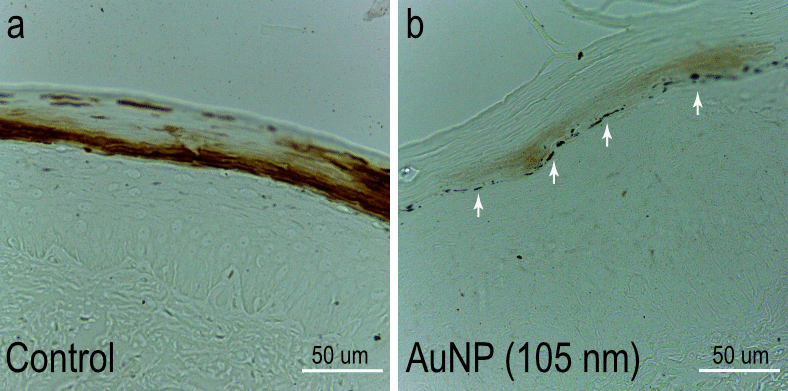
**

**Figure S6:** Representative phase-contrast images obtained from skin sections harvested from the hind-paw of rat that was topically exposed to AuNP (105 ± 11 nm). Sections were silver stained, and AuNP were detected as black deposits in the epidermal-dermal area (arrows). Silver stained skin section from naïve rat was used as control.
